# The interaction between age and parity on adverse pregnancy and neonatal outcomes

**DOI:** 10.3389/fmed.2023.1056064

**Published:** 2023-02-23

**Authors:** Jiayang Dai, Ya Shi, Yinshuang Wu, Lu Guo, Dan Lu, Ying Chen, Yuanyuan Wang, Hanpeng Lai, Xiang Kong

**Affiliations:** ^1^School of Nursing and School of Public Health, Yangzhou University, Yangzhou, China; ^2^Department of Obstetrics and Gynecology, Clinical Medical College, Yangzhou University, Yangzhou, Jiangsu Province, China; ^3^School of Health Sciences, Faculty of Biology, Medicine and Health, The University of Manchester, Manchester, United Kingdom; ^4^Graduate School of Dalian Medical University, Dalian, China; ^5^Institute of Translational Medicine, Medical College, Yangzhou University, Yangzhou, Jiangsu Province, China; ^6^Jiangsu Key Laboratory of Experimental & Translational Non-Coding RNA Research, Yangzhou, Jiangsu Province, China; ^7^Nanjing Stomatological Hospital, Medical School of Nanjing University, Nanjing, China

**Keywords:** retrospective cohort study, advanced maternal age, parity, pregnancy outcome, neonatal outcome

## Abstract

**Background:**

Although age and parity are recognized as associated factors for adverse pregnancy outcomes, there are no studies exploring the interaction between the two during pregnancy. This study aimed to investigate the impact of the interaction between age and parity on adverse pregnancy outcomes.

**Methods:**

This was a retrospective cohort study with 15,861 women aged ≥20 years. All women were grouped according to age, parity, and a mix of the two. The data were analyzed using multivariate logistic regression analysis.

**Results:**

Age, parity, and interaction between the two were related with the risk of gestational hypertension, eclampsia/pre-eclampsia, placenta previa, placental implantation, postpartum hemorrhage, preterm birth, cesarean section, and Apgar score <7 within 5 min of birth. The risk of gestational diabetes mellitus and transfer to the neonatal unit was linked with age and the interaction between age and parity, but the impact of parity was not statistically significant. The risk of anemia, placental abruption, premature rupture of the membrane, oligohydramnios, and macrosomia was only associated with parity; the risk of fetal distress was only associated with age.

**Conclusion:**

The interaction between advanced age and parity might results in more adverse outcomes for both puerpera and infants, necessitating additional prenatal screening and health education throughout pregnancy.

## Introduction

The International Federation of Gynaecology and Obstetrics (FIGO) defines advanced maternal age (AMA) as age ≥35 years at the time of expected delivery (1). At present, the definition of very advanced maternal age (vAMA) is rather debatable, with some researchers defining ≥40 years at the time of expected delivery as very advanced maternal age (vAMA) (2, 3).

Currently, international research indicated that the proportion of AMA and vAMA increased with year (4, 5). In China, studies have shown that the proportion of AMA increased from 7.4% in 2013 to 15.9% in 2018 (6). In addition, as assisted reproductive technology becomes more prevalent, the proportion of AMA is expected to increase in the coming years (7). There is a gradual increase in the number of AMA in numerous countries. According to a survey conducted by the World Health Organization (WHO) of 308,149 mothers and newborns covering 29 countries in Africa, Asia, Latin America, and the Middle East indicated that the proportion of AMA reached 12.3% (8). The Centers for Disease Control and Prevention (CDC) reported that the fertility rate for women aged 35–44 years increased from 19.8‰ in 1980 to 52.6‰ in 2018 (9).

There are several risk factors for adverse pregnancy outcomes, with advanced age and parity being the most significant (1, 10). Many studies have been conducted to investigate the relationship between advanced age or parity and adverse pregnancy outcomes (1, 11). It has been demonstrated that advanced maternal age is associated with numerous adverse pregnancy outcomes. Vandekerckhove discovered that the risk of maternal and fetal complications increased steadily with age and was particularly high after 35 years (12). Guarga Montori also discovered that women >35 years had worse perinatal outcomes than younger women, with the disparity being more pronounced in patients >40 (13). It is debatable if parity is a risk factor for adverse pregnancy outcomes. Shechter-Maor G indicated that pregnancy complications were much more likely in nulliparous women of advanced maternal age than multiparous women of the same age (14), and Schimmel MS also found similar conclusions (15). Muniro Z, on the other hand, discovered that grand multiparity was associated with increased risks of adverse pregnancy outcomes, such as postpartum hemorrhage, gestational hypertension, gestational diabetes mellitus, and high perinatal mortality (16). Therefore, the relationship between parity and adverse pregnancy outcomes remains to be studied. Advanced age and parity have been studied more frequently in relation to adverse pregnancy outcomes; nevertheless, to our knowledge, there has been no investigation into the impact of the interaction between advanced age and parity on adverse pregnancy outcomes and neonatal outcomes, which requires further investigation.

Overall, this study conducted retrospective analyzes on the interaction between age and parity on adverse pregnancy and neonatal outcomes to fill the gap in this area. We will identify trends in the risk of pregnancy outcomes and neonatal outcomes across age and parity, which will give obstetric healthcare professionals with more detailed clinical evidence for more informed clinical consultation and decision-making.

## Materials and methods

### Study population and design

The study population was women aged ≥20 years who had a singleton birth at the Northern Jiangsu People’s Hospital in Yangzhou City, Jiangsu Province, China, between January 2016 and December 2020.

The inclusion criteria were ≥28 weeks gestational week of delivery, age ≥20 years, and singleton live birth. The exclusion criteria were induction of labor, intrauterine fetal death, viral myocarditis, congenital heart disease, liver, kidney, lung, and other important organ pathologies, serious primary diseases, combined with serious infectious diseases, mental disorders, or cognitive dysfunction. Fifteen thousand eight hundred and sixty-one mothers met the criteria.

### Study methods

#### Data collection

The Northern Jiangsu People’s Hospital’s electronic information system was used to collect the data for this investigation. Two researchers independently collected case information of all participants (Collecting time: May 2022–July 2022), including age at delivery, height, pre-pregnancy body mass, education, residence, mode of conception, parity, mode of previous deliveries, and captured data on maternal pregnancy outcomes and neonatal outcomes *via* the electronic information system. They then checked each other’s work, and if a discrepancy was discovered, a third person reviewed the data.

#### Diagnostic criteria

All women were grouped according to three bases: age, parity, and a mixture of age and parity. According to age, pregnant women were divided into five groups: the first appropriate age group (20–24 years, A1 group), the second appropriate age group (25–29 years, A2 group), the third appropriate age group (30–34 years, A3 group), the advanced maternal age group (35–39 years old, AMA group), and the very advanced maternal age group (≥40 years, vAMA group). For parity, pregnant women were divided into two groups: a nulliparous group (parity = 1) and a multiparous group (parity ≥ 2). With regard to the mixture of age and parity, pregnant women were divided into 10 groups, combining the two previous groupings. Education was categorized into bachelor’s degree or above and no bachelor’s degree. Residence was divided into urban and rural areas. Marital status was divided into married and unmarried. Smoking was divided into Yes and No. Pre-pregnancy BMI was calculated by dividing pre-pregnancy weight (kg) by the square of height (m^2^). BMI was divided into four categories using Asian-specific cut-offs (17): <18.5, 18.5–23, 23–27.5, and ≥27.5 kg/m^2^. Gestational weight gain (GWG) was classified following the 2009 Institute of Medicine (IOM) guidelines, the standard divides GWG into Inadequate, Adequate, and Excessive according to different prenatal weight standards for pregnant women (18). Pregnancy was divided into two categories: assisted reproduction and natural conception. Gestational weeks were separated into three categories: 28–31, 32–36, and 37 weeks and above, with deliveries at less than 37 weeks being considered as preterm births. For multiparas, the form of the previous birth was classified into cesarean section and vaginal delivery.

The outcome indicators were maternal pregnancy outcomes and neonatal outcomes. Maternal pregnancy outcomes included gestational hypertension, eclampsia/pre-eclampsia, gestational diabetes mellitus (GDM), intrahepatic cholestasis of pregnancy (ICP), anemia, placenta previa, placental abruption, placental implantation, premature rupture of membranes, postpartum hemorrhage, oligohydramnios, preterm birth, and cesarean section. Neonatal outcomes included macrosomia, fetal distress, transfer to the neonatal unit, neonatal jaundice, and an Apgar score <7 within 5 min of birth. All outcome indicators were diagnosed according to the International Classification of Diseases 10th edition (ICD-10).

### Statistical analysis

Microsoft Excel 2007 was used to record and organize the data. The SPSS 26.0 statistical program was utilized for data collection and analysis. Quantitative data were described as $\bar{x}\;\pm \;s,$ and qualitative data were expressed as frequencies (percentage). The *χ*^2^ test was used for comparisons among groups. Single-factor analysis was used to compare the prevalence of adverse pregnancy outcomes and adverse neonatal outcomes of pregnant women in the single age group, single parity group, and mixed group. Significant factors identified by the single-factor analysis were incorporated into a multivariate logistic regression analysis. After adjusting for possible confounding factors, the adjusted *OR* (a*OR*) and 95% confidence interval (*95% CI*) were used to show the risk of pregnancy and neonatal adverse outcomes in the single age group, single parity group, and combination group. All *p* values were two-sided tests, with *p* < 0.05 indicating statistical significance.

### Ethical considerations

Due to the absence of an ethical statement component in our research, an ethics application is unnecessary. This data collection was approved by the Obstetrics and Gynecology Department of the Northern Jiangsu People’s Hospital.

## Results

### Sociodemographic data and pre-pregnancy characteristics

A total of 15,861, women were included in this study. Of these, 2,586 (16.3%) women were aged 20–24 years, 8,057 (50.80%) women were aged 25–29 years, 3,636 (22.92%) women were aged 30–34 years, 1,314 (8.28%) women were aged 35–39 years, and 268 (1.69%) women were aged ≥40 years. There were 12,002 nulliparous women (75.67%) and 3,859 multiparous women (24.33%). 50.11% of these women have a bachelor’s degree or above, 60.38% of women living in urban areas. 96.65% of women are married, and 1.08% of women have a bad habit of smoking. 34.81% of women had a pre-pregnancy BMI in the normal range, 3.35% of women weighed less than the normal range, and 61.85% of pregnant women weighed more than the normal range. 69.97% of women GWG at an appropriate level, 9.95% of women gained less gestational weight, and 20.08% of women gained more during pregnancy. In terms of mode of conception, 5.43% of women using assisted reproductive technologies. In terms of the gestational week of delivery, 88.21% of women gave birth at full term and 11.79% of women gave birth prematurely. 68.26% of women had a cesarean section at their previous birth, and 31.74% of women had a vaginal delivery (Table 1).

**Table 1:** Sociodemographic and pre-pregnancy characteristics of study participants.

Variables	Total	Nulliparas					*p*	Multiparas					*p*
		A1 (%)	A2 (%)	A3 (%)	AMA (%)	vAMA (%)		A1 (%)	A2 (%)	A3 (%)	AMA (%)	vAMA (%)	
*n*	15,861	2,541	7,821	1,374	230	36		45	236	2,262	1,084	232	
Education							0.00^***^						0.00^***^
Bachelor’s degree or above	7,948 (50.11)	1,240 (48.80)	4,536 (58.00)	721 (52.47)	70 (30.43)	10 (27.78)		7 (15.56)	32 (13.56)	898 (39.7)	375 (34.59)	59 (25.43)	
Undergraduate	7,913 (49.89)	1,301 (51.20)	3,285 (42.00)	653 (47.53)	160 (69.57)	26 (72.22)		38 (84.44)	204 (86.44)	1,364 (60.3)	709 (65.41)	173 (74.57)	
Place of residence							0.00^***^						0.00^***^
Urban	9,577 (60.38)	1,321 (51.99)	5,005 (63.99)	901 (65.57)	132 (57.39)	22 (61.11)		18 (40.00)	95 (40.25)	1,359 (60.01)	605 (55.81)	119 (51.29)	
Rural	6,284 (39.62)	1,220 (48.01)	2,816 (36.01)	473 (34.43)	98 (42.61)	14 (38.89)		27 (60.00)	141 (59.75)	903 (39.92)	479 (44.19)	113 (48.71)	
Marital status							0.92						0.85
Married	15,329 (96.65)	2,454 (96.58)	7,563 (96.70)	1,326 (96.51)	220 (95.65)	35 (97.22)		44 (97.78)	231 (97.88)	2,184 (96.55)	1,048 (96.68)	224 (96.55)	
Unmarried	532 (3.35)	87 (3.42)	258 (3.30)	48 (3.49)	10 (4.35)	1 (2.78)		1 (2.22)	5 (2.12)	78 (3.45)	36 (3.32)	8 (3.45)	
Smoking							0.89						0.95
Yes	172 (1.08)	28 (1.10)	82 (1.05)	15 (1.09)	2 (0.87)	1 (2.78)		1 (2.22)	3 (1.27)	26 (1.15)	12 (1.11)	2 (0.86)	
No	15,689 (98.92)	2,513 (98.90)	7,739 (98.95)	1,359 (98.91)	228 (99.13)	35 (97.22)		44 (97.78)	233 (98.73)	2,236 (98.85)	1,072 (98.89)	230 (99.14)	
Pre-pregnancy BMI (kg/m^2^)							0.07						0.72
<18.5	531 (3.35)	126 (4.96)	274 (3.50)	49 (3.57)	7 (3.04)	1 (2.78)		1 (2.22)	4 (1.69)	42 (2.92)	23 (1.20)	4 (1.72)	
18.5–23	5,521 (34.81)	890 (35.03)	2,660 (34.01)	470 (34.21)	89 (38.70)	14 (38.89)		18 (40)	92 (38.98)	823 (36.38)	398 (36.72)	67 (28.88)	
23–27.5	6,320 (39.85)	1,017 (40.02)	3,128 (39.99)	549 (39.96)	87 (37.83)	14 (38.89)		18 (40)	92 (38.98)	899 (39.74)	418 (38.56)	98 (42.24)	
≥27.5	3,489 (22.00)	508 (19.99)	1,759 (22.49)	306 (22.27)	47 (20.43)	7 (19.44)		8 (17.78)	48 (20.34)	498 (22.02)	245 (22.60)	63 (27.16)	
Gestational weight gain (GWG)							0.54						0.98
Inadequate	1,578 (9.95)	240 (9.45)	780 (9.97)	140 (10.19)	33 (14.35)	2 (5.56)		3 (6.67)	25 (10.59)	218 (9.64)	112 (10.33)	25 (10.78)	
Adequate	11,098 (69.97)	1,796 (70.68)	5,483 (70.11)	957 (69.65)	151 (65.65)	27 (75.00)		33 (73.33)	163 (69.07)	1,584 (70.03)	742 (68.45)	162 (69.83)	
Excessive	3,185 (20.08)	505 (19.87)	1,558 (19.92)	277 (20.16)	46 (20.00)	7 (19.44)		9 (20.00)	48 (20.34)	460 (20.34)	230 (21.22)	45 (19.40)	
Assisted reproduction							0.00^***^						0.00^***^
Yes	862 (5.43)	5 (0.20)	385 (4.92)	273 (19.87)	79 (34.35)	16 (44.44)		0	5 (2.12)	25 (1.11)	58 (5.35)	16 (6.90)	
No	14,999 (94.57)	2,536 (99.80)	7,436 (95.08)	1,101 (80.13)	151 (65.65)	20 (55.56)		45 (100)	231 (97.88)	2,237 (98.89)	1,026 (94.65)	216 (93.10)	
Week of pregnancy (weeks)							0.31						0.11
28–31 weeks	199 (1.25)	31 (1.22)	94 (1.20)	15 (1.09)	3 (1.30)	1 (2.78)		1 (2.22)	3 (1.27)	32 (1.41)	15 (1.38)	4 (1.72)	
32–36 weeks	1,671 (10.54)	255 (10.04)	780 (9.97)	122 (8.88)	34 (14.78)	5 (13.89)		6 (13.33)	30 (12.71)	244 (10.79)	159 (14.67)	36 (15.52)	
≥37 weeks	13,991 (88.21)	2,255 (88.74)	6,947 (88.82)	1,237 (90.03)	193 (83.91)	30 (83.33)		38 (84.44)	203 (86.02)	1,986 (87.8)	910 (83.95)	192 (82.76)	
Previous delivery													0.23
Cesarean section	2,634 (68.26)	——	——	——	——	——		28 (62.22)	170 (72.03)	1,526 (67.46)	759 (70.02)	151 (65.09)	
Vaginal delivery	1,225 (31.74)	——	——	——	——	——		17 (37.78)	66 (27.97)	736 (32.54)	325 (29.98)	81 (34.91)	

^*^p < 0.05, statistically difference, ^**^p < 0.01, statistically significant difference, and ^***^p < 0.001, highly statistically significant difference. A1: 20–24 years old women, A2: 25–29 years old women, A3: 30–34 years old women, AMA: 35–39 years old women, and vAMA: ≥40 years old women.

### Incidence of pregnancy outcomes and neonatal outcomes in women of different ages and parities

The incidence of adverse pregnancy outcomes ranged from 1.07 to 22.91%, with gestational diabetes mellitus (22.91%), premature rupture of membranes (17.92%), and transfer to the neonatal unit (14.48%) being the three most prevalent diseases.

The incidence of adverse pregnancy and neonatal outcomes for single age groups is shown in Table 2. In terms of a single age group, the difference in the following outcomes among the five age groups was statistically significant, including the prevalence of gestational hypertension, eclampsia/pre-eclampsia, gestational diabetes mellitus, placenta previa, placental implantation, postpartum hemorrhage, preterm birth, cesarean section, vaginal delivery, fetal distress, transfer to neonatal unit, and Apgar score <7 within 5 min of birth, while the difference in the remaining indices was not statistically significant.

**Table 2:** Incidences of adverse pregnancy outcomes and neonatal outcomes for the single age group.

Variables	Total	A1 (%)	A2 (%)	A3 (%)	AMA (%)	vAMA (%)	*p*
*n*	15,861	2,586	8,057	3,636	1,314	268	
Gestational hypertension	740 (4.67)	68 (2.63)	317 (3.93)	177 (4.87)	136 (10.35)	42 (15.67)	0.00^***^
Eclampsia/pre-eclampsia	537 (3.39)	31 (1.20)	230 (2.85)	146 (4.02)	97 (7.38)	33 (12.31)	0.00^***^
Gestational diabetes mellitus	3,633 (22.91)	481 (18.60)	1,736 (21.55)	883 (24.28)	436 (33.18)	97 (36.19)	0.00^***^
Intrahepatic cholestasis of pregnancy	374 (2.36)	59 (2.28)	201 (2.49)	77 (2.12)	29 (2.21)	8 (2.99)	0.70
Anemia	262 (1.65)	36 (1.39)	118 (1.46)	74 (2.04)	27 (2.05)	7 (2.61)	0.06
Placenta previa	279 (1.76)	20 (0.77)	81 (1.01)	80 (2.20)	77 (5.86)	21 (7.84)	0.00^***^
Placental abruption	174 (1.10)	27 (1.04)	83 (1.03)	43 (1.18)	17 (1.29)	4 (1.49)	0.83
Placental implantation	170 (1.07)	28 (1.08)	71 (0.88)	33 (0.91)	29 (2.21)	9 (3.36)	0.00^***^
Premature rupture of membrane	2,843 (17.92)	478 (18.48)	1,492 (18.52)	618 (17.00)	211 (16.06)	44 (16.42)	0.09
Postpartum hemorrhage	665 (4.19)	82 (3.17)	304 (3.77)	164 (4.51)	89 (6.77)	26 (9.70)	0.00^***^
Oligohydramnios	741 (4.67)	116 (4.49)	371 (4.60)	168 (4.62)	64 (4.87)	22 (8.21)	0.09
Preterm birth	1,870 (11.79)	293 (11.33)	907 (11.26)	413 (11.36)	211 (16.06)	46 (17.16)	0.00^***^
Cesarean section	8,878 (55.97)	1,123 (43.43)	4,133 (51.30)	2,338 (64.30)	1,063 (80.90)	221 (82.46)	0.00^***^
Macrosomia	549 (3.46)	90 (3.48)	258 (3.20)	137 (3.77)	55 (4.19)	9 (3.36)	0.32
Fetal distress	120 (0.76)	33 (1.28)	65 (0.81)	15 (0.41)	6 (0.46)	1 (0.37)	0.002^**^
Transfer to neonatal unit	2,296 (14.48)	309 (11.95)	1,120 (13.90)	540 (14.85)	256 (19.48)	71 (26.49)	0.00^***^
Neonatal jaundice	778 (4.91)	119 (4.60)	394 (4.89)	171 (4.70)	73 (5.56)	21 (7.84)	0.14
Apgar score <7 within 5 min of birth	278 (1.75)	33 (1.28)	127 (1.58)	72 (1.98)	36 (2.74)	10 (3.73)	0.00^***^

^*^p < 0.05, statistically difference, ^**^p < 0.01, statistically significant difference, and ^***^p < 0.001, highly statistically significant difference. A1: 20–24 years old women, A2: 25–29 years old women, A3: 30–34 years old women, AMA: 35–39 years old women, and vAMA: ≥40 years old women.

The incidence of adverse pregnancy outcomes and neonatal outcomes for single parity group are shown in Table 3. In terms of the single parity group, the difference in the following outcomes among the two parity groups was statistically significant, including the prevalence of gestational hypertension, eclampsia/pre-eclampsia, gestational diabetes mellitus, anemia, placenta previa, placental abruption, placental implantation, premature rupture of membranes, postpartum hemorrhage, oligohydramnios, preterm birth, cesarean section, macrosomia, fetal distress, transfer to the neonatal unit, and Apgar score <7 points within 5 min of birth, whereas the difference in the incidence of intrahepatic cholestasis of pregnancy and neonatal jaundice was not statistically significant.

**Table 3:** Incidences of pregnancy outcomes and neonatal outcomes for the single parity group.

Variables	Nulliparas (%)	Multiparas (%)	*p*
*n*	12,002	3,859	
Gestational hypertension	487 (4.06)	253 (6.56)	0.00^***^
Eclampsia/pre-eclampsia	349 (2.91)	188 (4.87)	0.00^***^
Gestational diabetes mellitus	2,627 (21.89)	1,006 (26.07)	0.00^***^
Intrahepatic cholestasis of pregnancy	296 (2.47)	78 (2.02)	0.11
Anemia	176 (1.47)	86 (2.23)	0.00^***^
Placenta previa	133 (1.11)	146 (3.78)	0.00^***^
Placental abruption	128 (1.07)	70 (1.81)	0.00^***^
Placental implantation	114 (0.95)	56 (1.45)	0.01^*^
Premature rupture of membrane	2,226 (18.55)	418 (10.83)	0.00^***^
Postpartum hemorrhage	452 (3.77)	213 (5.52)	0.00^***^
Oligohydramnios	946 (7.88)	103 (2.67)	0.00^***^
Preterm birth	1,340 (11.16)	530 (13.73)	0.00^***^
Cesarean section	6,056 (50.46)	2,822 (73.13)	0.00^***^
Macrosomia	393 (3.27)	156 (4.04)	0.02^*^
Fetal distress	107 (0.89)	13 (0.34)	0.00^***^
Transfer to neonatal unit	1,688 (14.06)	608 (15.76)	0.00^***^
Neonatal jaundice	573 (4.77)	205 (5.31)	0.18
Apgar score <7 within 5 min of birth	182 (1.52)	96 (2.49)	0.00^***^

^*^p < 0.05, statistically difference, ^**^p < 0.01, statistically significant difference, ^***^p < 0.001, highly statistically significant difference. A1: 20–24 years old women, A2: 25–29 years old women, A3: 30–34 years old women, AMA: 35–39 years old women, and vAMA: ≥40 years old women.

The incidence of pregnancy outcomes and neonatal outcomes for 10 groups of mixed age and parity are shown in Table 4. The difference in the following outcomes among these 10 groups was statistically significant: prevalence of gestational hypertension, eclampsia/pre-eclampsia, gestational diabetes mellitus, placenta previa, placental implantation, postpartum hemorrhage, preterm birth, cesarean section, transfer to neonatal unit, and Apgar score <7 points within 5 min of birth, while the difference in the remaining indices was not statistically significant.

**Table 4:** Incidences of adverse pregnancy outcomes and neonatal outcomes for the mixture group with different ages and parities.

Variables	Total (%)	Nulliparas					Multiparas					*p*
		A1 (%)	A2 (%)	A3 (%)	AMA (%)	vAMA (%)	A1 (%)	A2 (%)	A3 (%)	AMA (%)	vAMA (%)	
*n*	15,861	2,541	7,821	1,374	230	36	45	236	2,262	1,084	232	
Gestational hypertension	740 (4.67)	66 (2.60)	308 (3.94)	77 (5.60)	30 (13.04)	6 (16.67)	2 (4.44)	9 (3.81)	100 (4.42)	106 (9.78)	36 (15.52)	0.00^***^
Eclampsia/pre-eclampsia	537 (3.39)	30 (1.18)	223 (2.85)	72 (5.24)	20 (8.70)	4 (11.11)	1 (2.22)	7 (2.97)	74 (3.27)	77 (7.10)	29 (12.50)	0.00^***^
Gestational diabetes mellitus	3,633 (22.91)	474 (18.65)	1,689 (21.60)	362 (26.35)	88 (38.26)	14 (38.89)	7 (15.56)	47 (19.92)	521 (23.03)	348 (32.10)	83 (35.78)	0.00^***^
Intrahepatic cholestasis of pregnancy	374 (2.36)	58 (2.28)	196 (2.51)	35 (2.55)	5 (2.17)	2 (5.56)	1 (2.22)	5 (2.12)	42 (1.86)	24 (2.21)	6 (2.59)	0.80
Anemia	262 (1.65)	35 (1.38)	112 (1.43)	22 (1.60)	5 (2.17)	2 (5.56)	1 (2.22)	6 (2.54)	52 (2.30)	22 (2.03)	5 (2.16)	0.07
Placenta previa	279 (1.76)	20 (0.79)	77 (0.98)	23 (1.67)	10 (4.35)	3 (8.33)	0	4 (1.69)	57 (2.52)	67 (6.18)	18 (7.76)	0.00^***^
Placental abruption	174 (1.10)	26 (1.02)	80 (1.02)	14 (1.02)	3 (1.30)	1 (2.78)	1 (2.22)	3 (1.27)	29 (1.28)	14 (1.29)	3 (1.29)	0.95
Placental implantation	170 (1.07)	28 (1.10)	70 (0.90)	12 (0.87)	3 (1.30)	1 (2.78)	0	1 (0.42)	21 (0.93)	26 (2.40)	8 (3.45)	0.00^***^
Premature rupture of membrane	2,845 (17.94)	473 (18.61)	1,454 (18.59)	253 (18.41)	42 (18.26)	6 (16.67)	7 (15.56)	38 (16.10)	365 (16.14)	169 (15.59)	38 (16.38)	0.14
Postpartum hemorrhage	665 (4.19)	80 (3.15)	296 (3.78)	60 (4.37)	12 (5.22)	4 (11.11)	2 (4.44)	8 (3.39)	104 (4.60)	77 (7.10)	22 (9.48)	0.00^***^
Oligohydramnios	741 (4.67)	114 (4.49)	360 (4.60)	62 (4.51)	11 (4.78)	2 (5.56)	2 (4.44)	11 (4.66)	106 (4.69)	53 (4.89)	20 (8.62)	0.79
Preterm birth	1,870 (11.79)	286 (11.26)	874 (11.18)	137 (9.97)	37 (16.09)	6 (16.67)	7 (15.56)	33 (13.98)	276 (12.20)	174 (16.05)	40 (17.24)	0.00^***^
Cesarean section	8,878 (55.97)	1,102 (43.37)	3,980 (50.89)	749 (54.51)	194 (84.35)	31 (86.11)	21 (46.67)	153 (64.83)	1,589 (70.25)	869 (80.17)	190 (81.90)	0.00^***^
Macrosomia	549 (3.46)	88 (3.46)	250 (3.20)	48 (3.49)	7 (3.04)	0	2 (4.44)	8 (3.39)	89 (3.93)	48 (4.43)	9 (3.88)	0.55
Fetal distress	206 (1.30)	32 (1.26)	99 (1.270)	18 (1.31)	3 (1.30)	1 (2.78)	1 (2.22)	3 (1.27)	30 (1.33)	15 (1.38)	4 (1.72)	0.1
Transfer to neonatal unit	2,296 (14.48)	305 (12.00)	1,095 (14.00)	227 (16.52)	48 (20.87)	13 (36.11)	4 (8.89)	25 (10.59)	313 (13.84)	208 (19.19)	58 (25.00)	0.00^***^
Neonatal jaundice	778 (4.91)	117 (4.60)	382 (4.88)	62 (4.51)	11 (4.78)	1 (2.78)	2 (4.44)	12 (5.08)	109 (4.82)	62 (5.72)	20 (8.62)	0.37
Apgar score <7 within 5 min of birth	278 (1.75)	32 (1.26)	123 (1.57)	22 (1.60)	4 (1.74)	1 (2.78)	1 (2.22)	4 (1.69)	50 (2.21)	32 (2.95)	9 (3.88)	0.01^*^

^*^p < 0.05, statistically difference, ^**^p < 0.01, statistically significant difference, ^***^p < 0.001, highly statistically significant difference. A1: 20–24 years old women, A2: 25–29 years old women, A3: 30–34 years old women, AMA: 35–39 years old women, and vAMA: ≥ 40 years old women.

### Logistic regression analysis of adverse pregnancy outcomes and neonatal outcomes at different ages and parities

**Figure 1:**
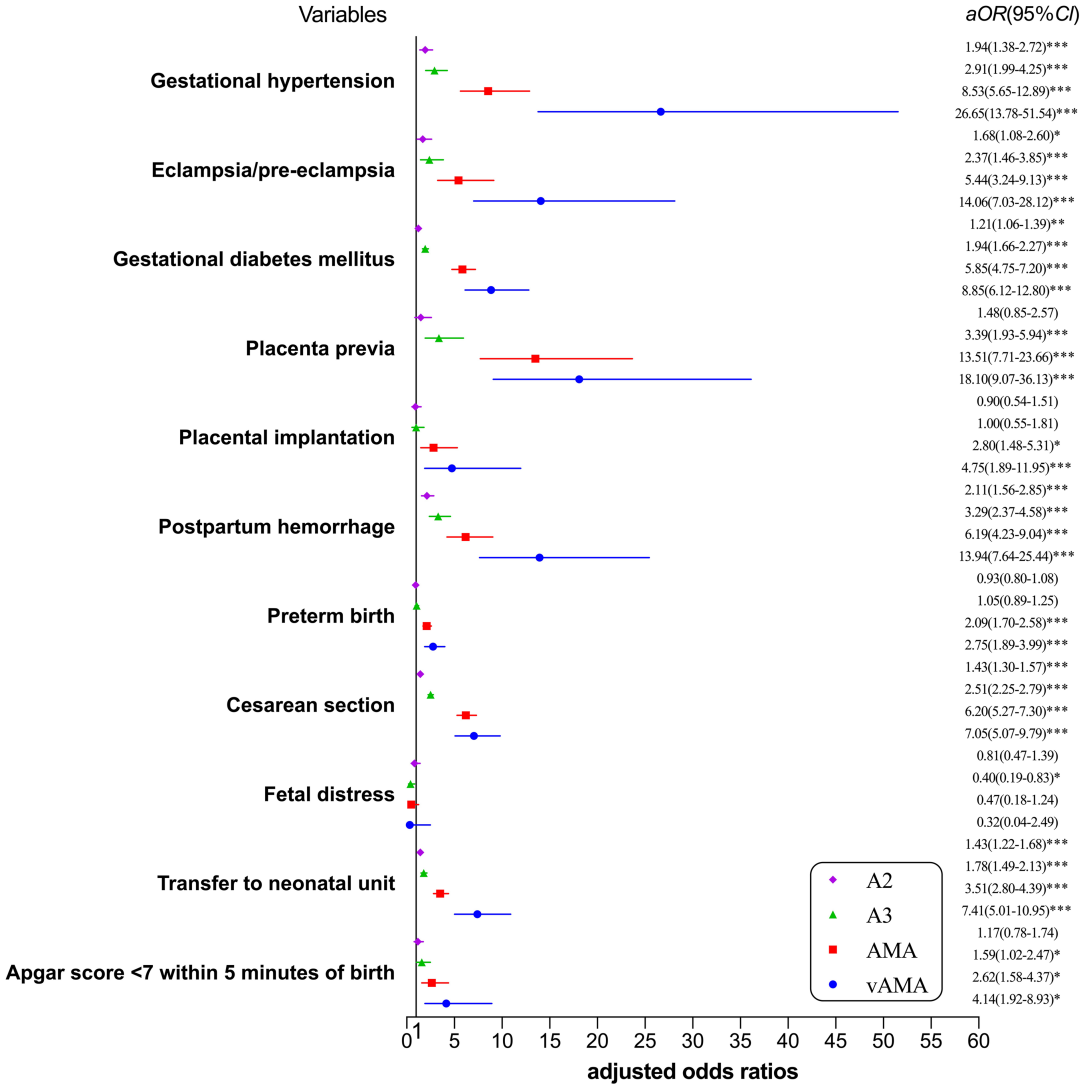
Forest plot for risk of adverse pregnancy outcomes and neonatal outcomes by single age group. ^*^p < 0.05, statistically difference, ^**^p < 0.01, statistically significant difference, and ^***^p < 0.001, highly statistically significant difference.

The risk of adverse pregnancy outcomes and neonatal outcomes for the single age group is shown in Figure 1. After correcting for confounding factors such as education, place of residence, pre-pregnancy BMI, assisted reproduction, and week of pregnancy, with increasing age, the risk of gestational hypertension, eclampsia/pre-eclampsia, gestational diabetes mellitus, placenta previa, placental implantation, postpartum hemorrhage, preterm birth, cesarean section, transfer to the neonatal unit, and Apgar score <7 within 5 min of birth showed an upward trend, while only the A3 group of women (30–34 years) had a reduced incidence of fetal distress.

**Figure 2:**
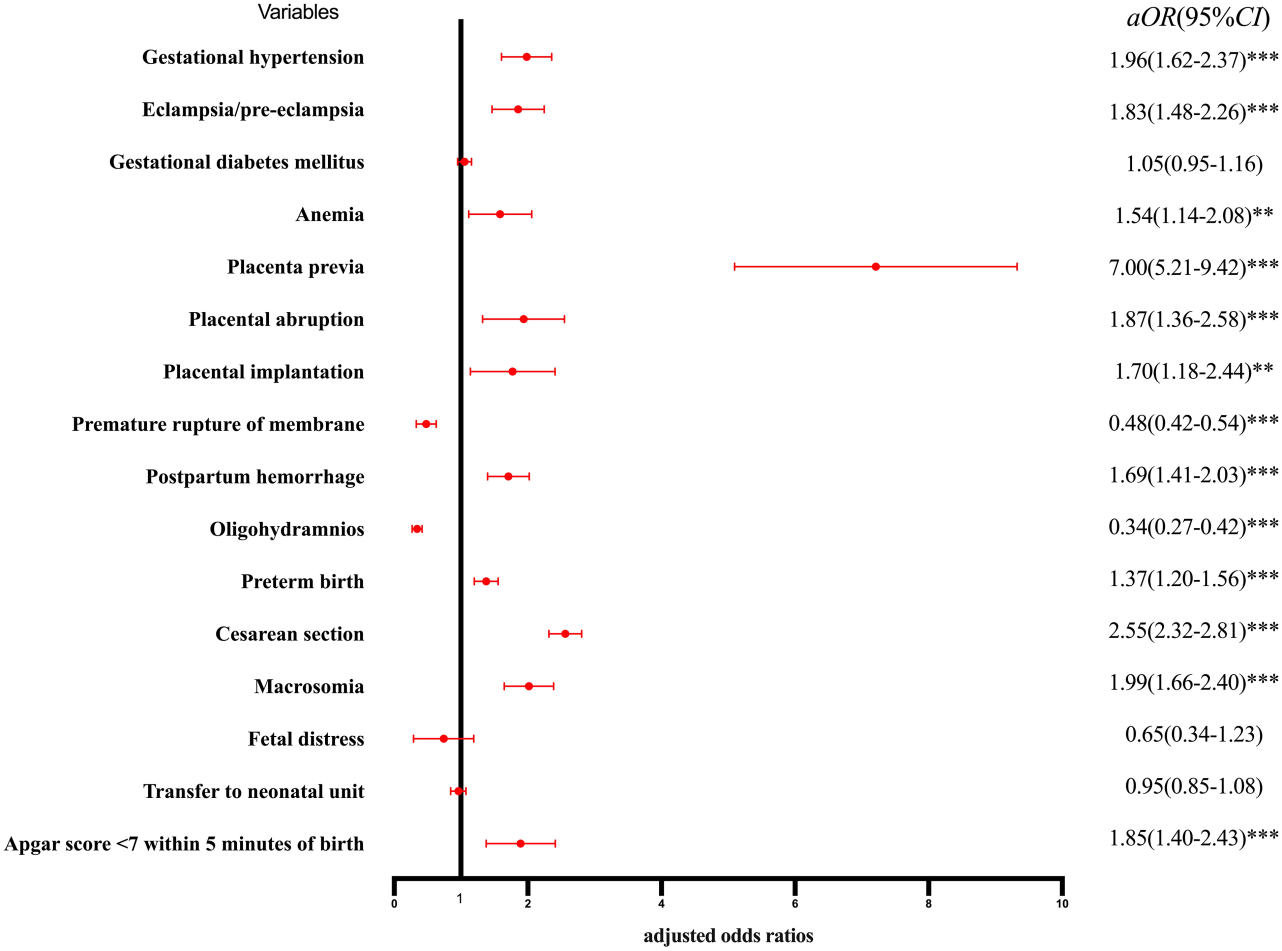
Forest plot for risk of adverse pregnancy outcomes and neonatal outcomes by single parity group. ^*^p < 0.05, statistically difference, ^*^p < 0.01, statistically significant difference, and ^***^p < 0.001, highly statistically significant difference.

The risk of adverse pregnancy outcomes and neonatal outcomes for the single parity group is shown in Figure 2. After correcting for confounding factors, such as education, place of residence, pre-pregnancy BMI, assisted reproduction, and week of pregnancy, multiparous women showed a higher risk of gestational hypertension, eclampsia/pre-eclampsia, anemia, placenta previa, placental abruption, placental implantation, postpartum hemorrhage, preterm birth, cesarean section, macrosomia, and Apgar score <7 within 5 min of birth than nulliparous women, whereas only the probability of premature rupture of membranes and oligohydramnios was less than that of nulliparous women.

**Figure 3:**
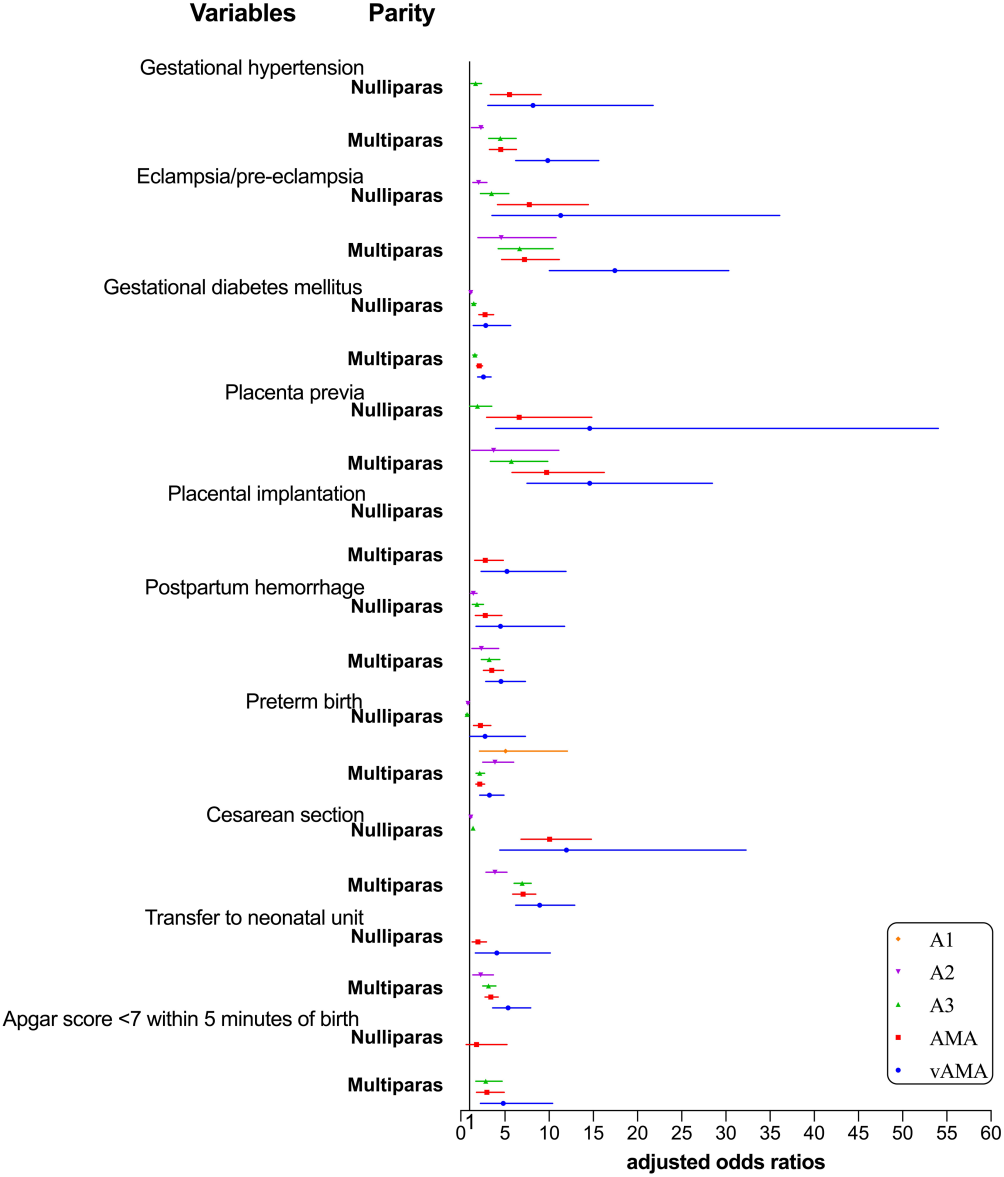
Forest plot for risk of adverse pregnancy outcomes and neonatal outcomes by group with different ages and parities.

The risk of adverse pregnancy outcomes and neonatal outcomes for the combination group with mixed age and parity is shown in Table 5 and Figure 3. The 20–24-year-old nulliparous women were taken as the control group, after correcting for confounding factors such as education, place of residence, and assisted reproduction. Regarding age, mothers with increasing age showed an increased risk of gestational hypertension, eclampsia/pre-eclampsia, gestational diabetes, placenta previa, placenta implantation, postpartum hemorrhage, cesarean section, transfer to the neonatal unit, and Apgar score <7 within 5 min of birth. From the perspective of parity, multiparous women showed a higher risk of placental previa, postpartum hemorrhage, transfer to the neonatal unit, and Apgar score <7 within 5 min of birth than nulliparous women of the same age. Only the risk of nulliparous women with AMA suffering from gestational hypertension, eclampsia/pre-eclampsia, gestational diabetes mellitus, and cesarean section and the risk of nulliparous women with vAMA suffering from gestational diabetes and cesarean section were greater than those of multiparous women of the same age; however, the risk of preterm birth did not vary regularly with age and parity.

**Table 5:** Multivariable adjusted odds ratios (*aOR*) for adverse pregnancy outcomes and neonatal outcomes by group with different ages and parities.

Variables	Parity	A1	A2	A3	AMA	vAMA
Gestational hypertension	Nulliparas	1	1.25 (0.95–1.64)	1.67 (1.17–2.37)^**^	5.52 (3.36–9.09)^***^	8.17 (3.07–21.76)^***^
	Multiparas	3.86 (0.89–16.84)	2.54 (1.22–5.29)^*^	4.47 (3.18–6.27)^***^	4.53 (3.26–6.29)^***^	9.86 (6.22–15.62)^***^
Eclampsia/pre-eclampsia	Nulliparas	1	2.02 (1.37–2.97)^***^	3.48 (2.22–5.45)^***^	7.75 (4.16–14.44)^***^	11.30 (3.54–36.08)^***^
	Multiparas	4.41 (0.58–33.80)	4.59 (1.95–10.78)^***^	6.66 (4.25–10.44)^***^	7.20 (4.64–11.15)^***^	17.43 (10.02–30.33)^***^
Gestational diabetes mellitus	Nulliparas	1	1.13 (1.00–1.26)^*^	1.47 (1.25–1.73)^***^	2.76 (2.06–3.71)^***^	2.84 (1.43–5.65)^**^
	Multiparas	0.92(0.41–2.08)	1.21 (0.87–1.70)	1.60 (1.38–1.85)^***^	2.10 (1.78–2.47)^***^	2.56 (1.92–3.43)^***^
Placenta previa	Nulliparas	1	1.08 (0.66–1.78)	1.88 (1.01–3.50)^*^	6.60 (2.94–14.82)^***^	14.60 (3.95–54.04)^***^
	Multiparas	0.00	3.71 (1.24–11.11)^*^	5.73 (3.34–9.84)^***^	9.71 (5.81–16.24)^***^	14.60 (7.49–28.48)^***^
Placental implantation	Nulliparas	0.68 (0.43–1.06)	0.67 (0.33–1.36)	1.47 (0.43–5.09)	3.82 (0.47–30.78)	0.68 (0.43–1.06)
	Multiparas	0.00	0.89 (0.12–6.67)	1.72 (0.90–3.26)	2.77 (1.59–4.80)^***^	5.24 (2.31–11.91)^***^
Postpartum hemorrhage	Nulliparas	1	1.41 (1.08–1.83)^*^	1.84 (1.33–2.56)^***^	2.78 (1.66–4.65)^***^	4.50 (1.73–11.75)^**^
	Multiparas	1.85 (0.43–8.02)	2.33 (1.27–4.29)^**^	3.22 (2.34–4.43)^***^	3.52 (2.56–4.83)^***^	4.55 (2.83–7.33)^***^
Preterm birth	Nulliparas	1	0.81 (0.70–0.94)^**^	0.73 (0.57–0.92)^**^	2.23 (1.46–3.40)^***^	2.74 (1.03–7.31)^*^
	Multiparas	5.07 (2.13–12.06)^***^	3.87 (2.50–5.99)^***^	2.16 (1.74–2.69)^***^	2.15 (1.72–2.69)^***^	3.25 (2.16–4.88)^***^
Cesarean section	Nulliparas	1	1.14 (1.03–1.26)^*^	1.39 (1.20–1.61)^***^	10.05 (6.83–14.79)^***^	11.95 (4.42–32.27)^***^
	Multiparas	1.74 (0.91–3.30)	3.87 (2.86–5.23)^***^	6.94 (6.05–7.96)^***^	7.06 (5.88–8.48)^***^	8.94 (6.21–12.89)^***^
Transfer to neonatal unit	Nulliparas	1	0.89 (0.77–1.03)	0.92 (0.74–1.14)	1.94 (1.28–2.92)^**^	4.09 (1.65–10.13)^**^
	Multiparas	1.34 (0.30–5.89)	2.25 (1.37–3.69)^**^	3.14 (2.49–3.97)^***^	3.41 (2.74–4.24)^***^	5.35 (3.62–7.93)^***^
Apgar score <7 within 5 min of birth	Nulliparas	1	1.08 (0.73–1.61)	1.15 (0.65–2.04)	1.78 (0.61–5.24)^*^	3.25 (0.42–25.42)
	Multiparas	4.21 (0.55–32.13)	2.78 (0.96–8.09)	2.83 (1.71–4.68)^*^	2.96 (1.79–4.91)^*^	4.82 (2.24–10.40)^*^

^*^p < 0.05, statistically difference, ^**^p < 0.01, statistically significant difference, ^***^p < 0.001, highly statistically significant difference. A1: 20–24 years old women, A2: 25–29 years old women, A3: 30–34 years old women, AMA: 35–39 years old women, and vAMA: ≥40 years old women.

## Discussion

Our study discovered that advanced age and multiple parities led to adverse pregnancy outcomes, while the interaction between advanced age and multiple parities further increased the risk of these outcomes. The relationship between different pregnancy outcomes and the three categories of age, parity, and the interaction between age and parity are not entirely consistent. According to our findings, the risk of gestational hypertension, eclampsia/pre-eclampsia, placenta previa, placental implantation, postpartum hemorrhage, preterm birth, cesarean section, and Apgar score <7 within 5 min of birth was associated with age, parity, and the interaction between the two. The risk of gestational diabetes mellitus and transfer to the neonatal unit was associated with age and the interaction between age and parity, but the impact of parity was not statistically significant; the risk of anemia, placental abruption, premature rupture of membranes, oligohydramnios, and macrosomia was only associated with parity; the risk of fetal distress was only associated with age.

We discovered that the risk of placenta previa, placental implantation, postpartum hemorrhage, and Apgar score <7 within 5 min of birth increased with age and parity, and that the interaction between age and parity enhanced the risk of these adverse outcomes even further. Both placenta previa and placental implantation are frequent placental abnormalities in mothers. A previous meta-analysis showed that AMA were 3.16 times more likely to have placenta previa than women of normal age (19). According to Ozdemirci’s study, parity increased the chance of placenta previa (20). Consistent with our findings, another study found that women with a history of numerous cesarean procedures had a higher likelihood of placental implantation (21). Our study further proved that the interaction between age and parity had a negative influence placental disease, possibly due to the reduced physiological function of the placenta in women who are older and have more parities and due to the history of poor childbirth in some women, such as multiple miscarriages and short intervals between cesarean sections, which can lead to adverse outcomes such as placenta previa and placental implantation. Placental problems predispose women to postpartum hemorrhage. Guarga demonstrated that AMA and vAMA were 1.13 times and 1.85 times more likely to have postpartum hemorrhage than those at an appropriate age (13). According to Ozdemirci’s study, parity increases the likelihood of placenta previa, which, in turn, increases the risk of postpartum hemorrhage (20). Our study also confirmed that the interaction between age and parity further increased the risk of postpartum hemorrhage, probably due to a prolonged third stage of labor or incomplete delivery of the placenta as a result of decreased placental function, which predisposes patients to postpartum hemorrhage. An Apgar score <7 within 5 min of birth is a common adverse neonatal outcome. The study by Mehari MA revealed that the risk of an Apgar score <7 within 5 min of birth was 7.51 times higher in older mothers than in those of appropriate age (22). There are few articles examining the relationship between an Apgar score <7 within 5 min of birth and parity, but our analysis revealed that either age or parity was a risk factor, and that the interaction between age and parity also increased its risk.

The interaction between age and parity further increased the risk of partial pregnancy outcomes; however, it was not very regular on some pregnancy outcomes. Gestational hypertension (HDP) is a leading global cause of maternal morbidity and mortality (23). Gestational hypertension includes gestational hypertension, pre-eclampsia, eclampsia, and chronic hypertension in pregnancy and chronic hypertension complicated by pre-eclampsia, which predispose mothers to increased vascular endothelial damage with age, inflammatory immune hyperactivation, and uteroplacental, resulting in an increase in blood pressure, systemic small artery spasm, a decrease in blood flow to the uterus and placenta, placental imbalance, and even placental abruption. Kahveci B observed that the prevalence of gestational hypertension in AMA was 1.55 times higher than that in women of normal age and that the prevalence increased with age, with the prevalence in vAMA being 1.68 times higher than that in women of normal age (2). The prevalence of eclampsia and pre-eclampsia was 2.39 and 9.92 times greater in the vAMA group compared to the normal age group, respectively. In terms of the relationship between gestational hypertension and age or parity, our findings are consistent with previous studies indicating that either age or parity was a risk factor for gestational hypertension. However, in terms of the interaction between age and parity, our result showed that the prevalence of nulliparous women in the AMA group was greater than that of multiparous women, which was inconsistent with previous studies and may be due to weak awareness of blood pressure control in nulliparous women of AMA. The cesarean section is a negative pregnancy outcome that has received increased attention in recent years. Our study identified age and parity as risk factors for cesarean section. However, in terms of the interaction between age and parity on cesarean section, the risk of cesarean section was higher in multiparous women of appropriate age than in nulliparous women of appropriate age, but it was higher in nulliparous women in the AMA and vAMA groups than in multiparous women. The risk of cesarean section was significantly elevated in nulliparous women of AMA; recent research suggests that this is likely attributable to faster changes in the risk of adverse outcomes in nulliparous women of AMA and an increased proportion of elective maternal cesarean section (24). Regarding preterm birth, the pathophysiology is still poorly known and may involve infection, hemorrhage, and maternal-fetal stress. Previous studies have demonstrated that the relationship between preterm birth and age follows a U-shaped curve, with the lowest risk at 30–34 years and increased risk at both younger than 24 years and older than 40 years (25). In the single age group of our study, we found a greater risk of preterm birth in advanced and very advanced maternal age than in appropriate age women, and in the single parity group, we found a greater risk of preterm birth in multiparous women than in nulliparous women, which is consistent with previous findings, but in the mixture of age and parity group, we found no regular relationship between the risk of preterm birth and changes in age and parity, possibly due to the interference of additional confounding factors. Therefore, the impact of the interaction between age and parity on the risk of preterm birth remains inconclusive.

Moreover, the risk of gestational diabetes mellitus and transfer to the neonatal unit was associated with age and the interaction between age and parity, according to our study. Gestational diabetes mellitus was the most prevalent condition among AMA in this study, and multiple investigations indicate the independent relationship between AMA and gestational diabetes mellitus (7, 26). Regarding the interaction between age and parity, our results showed that the prevalence of gestational diabetes mellitus increased with age, with a greater prevalence of multiparous women in the age-appropriate group than in the nulliparous age group. However, nulliparous women in the AMA group and vAMA group showed a higher risk of gestational diabetes mellitus than multiparous women in the AMA group and vAMA group, similar with the findings of Kahveci B and possibly attributable to abnormal glucose and lipid metabolism in advanced maternal age (27). Our study found no correlation between gestational diabetes mellitus and parity. Casagrande SS demonstrated that the risk of gestational diabetes mellitus was 1.57 times higher in women with ≥4 parities than in nulliparous women (28). In terms of neonatal outcomes, transferring to the neonatal unit is an adverse neonatal outcome that has been less well studied. Similar to our findings, Vandekerckhove’s study revealed an increased risk of fetal transfer to the neonatal unit in older women (12). Our study also revealed that the interaction between age and parity resulted in a higher rate of transfer to the neonatal unit in multiparous women than in nulliparous women.

In addition, the risk of certain pregnancy outcomes in this study was solely connected with a single factor. We found that the risk of anemia, placenta abruption, premature rupture of membrane, oligohydramnios, and macrosomia was exclusively connected with parity, while the risk of fetal distress was only associated with age. More research on anemia in pregnancy has been conducted in developing nations, likely because the majority of anemia in pregnancy is connected with maternal malnutrition. Lebso’s study showed that parity was a risk factor for anemia in pregnancy (29), and our study came to a similar conclusion. Lin’s study indicated that the risk of anemia in AMA is 1.386 times higher than that of women of appropriate age (30), but our study did not find a relationship between anemia and age or anemia and the interaction between age and parity, likely because our study population involved women who gave birth in tertiary care hospitals in a developed province in China. Future research should focus on anemic women residing in different locations and hospital levels. Placenta abruption and premature rupture of the membrane are common placental problems during pregnancy. Numerous studies have shown the relationship between placental abruption and parity, especially in women with previous cesarean sections, and a previous meta-analysis confirmed that advanced age was also a risk factor for placental abruption (19). Our study merely confirmed the link between placental abruption and parity, while the etiology of placental abruption is still poorly known. Premature rupture of membranes was one of the few pregnancy outcomes in our study, for which the risk was higher in nulliparous women than in multiparous women. It emerges from intricate, multiple pathways and predisposes women to premature births (31). Claramonte Nieto et al. found that the risk of premature rupture of membranes was 1.25 times higher in AMA than in women of appropriate age (32). However, neither age nor the interaction between age and parity were found to be related to premature membrane rupture. In addition, few studies have verified the relationship between oligohydramnios and age or parity; our study only verified that parity was a risk factor for oligohydramnios, possibly due to decreased placental function in multiparous women. Maternal hyperglycemia stimulates the secretion of insulin in large quantities, resulting in faster fetal growth and the formation of macrosomia (33). Lei showed that the risk of macrosomia in multiparous women was 1.26 times that of nulliparous women (34), which is similar to our findings. A Brazilian study showed that the odds of macrosomia in AMA were 1.22 times higher than in the appropriate age group (35), but our study did not find a relationship between macrosomia and age, and we did not find influence of the interaction between age and parity on macrosomia, which may be due to the low prevalence of macrosomia in our cohort. Fetal distress is a common adverse neonatal outcome. A Chinese study showed that the incidence of fetal distress increased in pregnant women >45 years old (36). The combination of gestational hypertensive disease in AMA results in changes in the small systemic vascular arteries and impaired circulation to the uterus and placenta, which leads to an inadequate supply of oxygen and nutrients to the fetus, thereby adversely affecting normal fetal development and even stillbirth. This leads to a lack of oxygen and nutrient supply to the fetus, thus adversely leading to fetal distress. Currently, the connection between fetal distress and advanced age is still controversial. Our study found that the incidence of fetal distress at 30–34 years old was reduced, and the incidence of other ages and parities was not statistically significant. This may be due to the low incidence of fetal distress in the cohort, and future cohort studies with bigger samples could be performed to determine the association between fetal distress and age and parity.

## Conclusion

This study focused on exploring the impact of the interaction between age and parity on adverse pregnancy and neonatal outcomes, which may fill the current gap in the mixed role of advanced age and parity on pregnancy outcomes. The following are the paper’s strengths: large sample size, 15,861 maternal cases collected from tertiary care hospitals over 5 years; wide distribution of maternal age, maternal age ≥20 years, including very advanced maternal age ≥40 years; detailed grouping based on three levels (age, parity, age, and parity); comprehensive comparison of the interaction between age and parity on adverse pregnancy outcomes and neonatal outcomes; comprehensive adverse outcomes, including neonatal jaundice, transfer to neonatal unit, and other adverse outcomes investigated less frequently in the past; comprehensive confounding factors include pre-pregnancy BMI, gestational weight gain and assisted reproduction, etc. The limitations are that the data were only from one tertiary care hospital in Yangzhou, China, which may be subject to selection bias and not representative of all advanced maternal age in China due to geographical differences, economic level differences, etc. In addition, due to the limitations of data administration in Chinese hospitals and the secrecy of certain topics, such as personal income, certain confounding factors, such as a family history of illness, medications taken during pregnancy, etc., may be excluded. There is still an increasing trend of advanced maternal age and very advanced maternal age, so a multicenter large sample study could be designed to further investigate the current status of pregnancy and the risk of adverse pregnancy outcomes among women with different ages and parities. In our study, the interaction between age and parity on adverse pregnancy outcomes such as intrahepatic cholestasis of pregnancy, preterm birth, and anemia was not clear, and further studies should be conducted to investigate these pregnancy outcomes. On the whole, all these results will provide clinicians, midwives, and obstetric nurses with more detailed information on the risk of adverse maternal outcomes and how to safeguard the health of the mother and fetus.

## Data availability statement

The data analyzed in this study are subject to the following licenses/restrictions: Our data are derived from the obstetrics and gynecology system of Northern Jiangsu People’s Hospital in Yangzhou, China, and cannot be disclosed to the public due to the confidentiality of personal data. Requests to access these datasets should be directed to Jiayang Dai, DjyYzu@163.com.

## Author contributions

JD and YS contributed to the conception or design of the study, and drafted the manuscript. JD, YiW, YC, and LG collected data and conducted data analysis. DL, YuW, and HL reviewed the literature and conducted some data analysis. JD, YS, XK, and DL revised the article and provided final approval of the manuscript. All authors contributed to the article and approved the submitted version.

## Conflict of interest

The authors declare that the research was conducted in the absence of any commercial or financial relationships that could be construed as a potential conflict of interest.

## Publisher’s note

All claims expressed in this article are solely those of the authors and do not necessarily represent those of their affiliated organizations, or those of the publisher, the editors and the reviewers. Any product that may be evaluated in this article, or claim that may be made by its manufacturer, is not guaranteed or endorsed by the publisher.
